# Alteration in amyloid β42, phosphorylated tau protein, interleukin 6, and acetylcholine during diabetes-accelerated memory dysfunction in diabetic rats: correlation of amyloid β42 with changes in glucose metabolism

**DOI:** 10.1186/s12993-015-0069-5

**Published:** 2015-08-14

**Authors:** You Zhou, Ying Zhao, Hailong Xie, Yan Wang, Lin Liu, Xinjia Yan

**Affiliations:** College of Pharmacy, Harbin University of Commerce, Harbin, Heilongjiang 150076 China; College of Pharmacy, Heilongjiang University of Chinese Medicine, Harbin, Heilongjiang 150040 China

**Keywords:** Diabetes mellitus, Cognitive impairment, Biomarkers, Cerebrospinal fluid

## Abstract

**Background:**

Diabetes accelerates memory dysfunction in a continuous, slowly pathological process. Studies suggest that the time course of certain biomarkers can characterize the pathological course of the disease to provide information for early intervention. Thus, there is an urgent need for validated biomarkers to characterize the cognitive impairment induced by DM. We aimed to detect changes in cerebrospinal fluid biomarkers such as amyloid β42, phosphorylated tau protein, interleukin 6, and acetylcholine in diabetic rats over time, and to analyse the relationship between diabetes and cognitive impairment.

**Methods:**

Rats were injected once intraperitoneally with 1% of streptozotocin to establish a diabetic model. Index changes were investigated longitudinally and all were measured at the end of the experiment at day 75. Aβ42, P-tau, IL-6, and ACh levels in CSF, insulin levels in plasma, and Aβ42 levels in plasma and brain tissue were measured by ELISA.

**Results:**

Compared with control, the diabetic model showed ACh in CSF to be decreased by day 15, continuing lower out to day 75. Aβ42 changes in brain and blood showed the same trends but exhibited minima at different time points: day 30 in CSF and day 15 in plasma. After the minimum, Aβ42 in cerebrospinal fluid rose and levelled off lower than in the control group, whereas Aβ42 in plasma rose and went above the controls at day 30, slowly trending upwards for the remainder of the experiment. P-tau protein in CSF in diabetic rats showed an increasing trend, becoming significantly different from the controls at day 60 and day 75. Aβ42 in CSF was strongly negatively correlated with blood glucose at day 15 and was negatively correlated with insulin in serum, particularly at day 45.

**Conclusion:**

Our longitudinal research model suggest that changes in the measured biomarkers appear before learning and memory impairments do. Aβ42 and ACh in the diabetes model group clearly changed from day 0 to day 45, and then P-tau and IL-6 varied significantly from day 45 to day 75. The reduced ACh levels observed possibly correlated with the factors common to changes in Aβ42, P-tau, and IL-6.

## Background

In recent years, the incidence of diabetes mellitus (DM) has been rising at an alarming rate. It has been reported that in China in 2010, the number of DM patients reached 114 million, accounting for 11.6% of China’s adult population and about one-third of total cases worldwide [1]. In research from the Netherlands, an increased risk of dementia was observed in patients suffering from type 2 diabetes [2]. Many studies have demonstrated a sequence of changes in neuropsychological and neurological behaviour in patients with diabetes [3, 4], and cognitive impairment with an increased risk of dementia in DM patients has become a serious social problem [5]. Increasing numbers of studies show that DM is an independent risk factor for Alzheimer disease (AD), vascular dementia, and other diseases closely related to AD. The mechanism of vascular dementia involves impairment of the insulin signalling pathway, altered glucose metabolism, metabolic changes such as amyloid β42 (Aβ42) imbalance, phosphorylation of tau protein (P-tau), and release of inflammatory cytokines (e.g. interleukin 6). These changes cause neuronal apoptosis, neuronal damage (particularly in the hippocampus), structural and functional synaptic damage, and neurotransmitter under-expression, collectively leading to cognitive impairment [6].

However, several questions remain concerning the cognitive impairment induced by DM: (1) Is the pathogenesis of the impairment caused by a sequence of phases, e.g. insulin signalling pathway damage, then impaired glucose metabolism, then inflammation? To understand this process, it is urgent to track the changes over time, particularly in insulin, Aβ42, tau protein, inflammatory cytokines, and other biological markers, to investigate the significance for pathogenesis of the intrinsic links among these. (2) What is the time dependence of the mechanism of damage as revealed by biomarkers, and of the resulting learning and memory deficit? The latter question has motivated research into preventative drug intervention in the hope of revealing the best time course of pharmacotherapy. Such research also uncovered the prevalence of blindness in DM.

Considerable evidence shows that diabetes accelerates memory dysfunction in a continuous, slowly pathological process. The incidence is difficult to assess, separate pathological stages are difficult to delineate clearly, and the injury mechanisms predominating at different times during this process continue to require clarification, all of which has hindered research and the formulation of optimal drug intervention strategies. A recent study in the United States suggests that the time course of certain biomarkers can characterize the pathological course of the disease to provide information for early intervention [7]. Thus, there is an urgent need for validated biomarkers to characterize the cognitive impairment induced by DM. It may be possible to interfere with the trajectory of the targeted biological markers in different phases of pathogenesis, and thus to provide the starting point for intervention in the disease process to prevent further damage. Studies have shown that during diabetes-induced cognitive impairment, metabolic and biochemical changes occur in the brain earlier than changes in function or structure. The metabolic changes comprise imbalances between amyloid β (Aβ) production and clearance, abnormal phosphorylation of tau protein, altered IL-6 levels (also associated with estimated lifetime cognitive decline [8]), lack of neurotransmitters, and structural and functional damage to neurons, ultimately leading to cognitive dysfunction. Both the learning and memory impairment, which influence each other, and the metabolic changes induced by DM causing damage to neurons (as evidenced by structural and functional studies), are progressive. In light of our previous results, we put forward the following hypothesis: ‘Changes in brain Aβ, P-tau protein, IL-6, and ACh are the first early events in the memory impairment process, and after a delay, which indicates the intervention time window, may cause the cognitive impairment observed in DM’. It is necessary to establish that the brain damage in DM is secondary to ensure that delaying or preventing the earliest pathological and functional changes will be effective. However, continuous dynamic multi-marker locus studies on cognitive impairment in diabetes have not yet been reported. Therefore, this study aimed to chart the time course of the biomarker changes—the initial memory impairment events—during the latency period of DM-induced cognitive impairment. The diabetes model was the streptozotocin (STZ)-injected rat, the biomarkers chosen for study were the CSF levels of Aβ42, P-tau, IL-6, and ACh, and the behavioural assessment method for learning and memory ability was the eight-arm maze test. We also looked at blood chemistry to obtain a better view of the entire pathological process, thereby providing a better understand of the stages of cognitive impairment induced by DM and experimental evidence to guide drug intervention. The study will also provide further evidence bearing on whether DM causes cognitive impairment.

## Methods

### Experimental animals

Male Wistar rats, 8–10 weeks old, weighing 200 ± 20 g, were obtained from the Beijing Vital River Laboratory Animal Technology Co., license number SCXK (Beijing) 2012-0001. The animal groups were housed separately in a 12-h alternating light–dark environment, with a temperature of 18–21°C and relative humidity of 50 ± 5%. The experimental protocol was approved by the Animal Ethics Committee of the Harbin University of Commerce and was carried out in accordance with the Guidance Suggestions for the Care and Use of Laboratory Animals, issued by the Ministry of Science and Technology of China [9].

### Diabetes model

Before the experiment, the animals were fasted for 12 h with water freely available. STZ stock solution was freshly prepared by dissolving STZ in citrate–sodium citrate buffer on an ice bath with protection from light, to give a 1% solution (pH 4.2–4.4). To establish the diabetes model, we administered an intraperitoneal injection of STZ stock solution at a dose of 55 mg/kg for the model group (n = 50), completing the injection within 10 min. We injected citric acid–sodium citrate buffer at the same volume (5.5 mL/kg) for the control group (n = 12). Seventy-two hours after injection, blood glucose was determined using a kit (Built Technology Co., Ltd., Nanjing, China), and animals with a fasting plasma glucose ≥16.7 mmol/L [10] were selected for inclusion in the experiment.

### Cerebrospinal fluid dynamic acquisition

CSF was continuously collected via the cisterna magna using a slightly modified version of a published procedure [11]. In a one-time procedure for a given animal, an intravenous infusion needle was slowly inserted to reach the cerebellar cistern, and the other end was connected to a 1-mL syringe, permitting slow collection of cerebrospinal fluid. After animal surgery, the wound was closed and the animals placed singly in cages for 5–7 days for wound healing. Once the diabetes model was established, CSF was collected every 15 days from each rat.

### Determination of Aβ42, P-tau, IL-6, and ACh in CSF, and Aβ42 content in brain tissue by ELISA

The Aβ42, P-tau, IL-6, and ACh contents of CSF were determined using ELISA kits (Bluegene Biotech Co., Shanghai, China). The microplate absorbance was measured at 450 nm. Diabetic rat brain tissues were taken at d75 and the homogenate centrifuged (15,000 rpm, 10 min, 4°C, Sigma 3K30). Aβ42 level in the supernatant was measured by ELISA. A kit based on the Bradford method (Nanjing Jiancheng Technology Co., Ltd., Nanjing, China) was used to measure tissue protein content. The Aβ42 content of brain tissue was expressed as picograms per milligram of total protein.

### Learning and memory ability of rats determined by the eight-arm maze test

After the diabetic rat model had been established for 60 days, learning and memory were tested by the eight-arm radial maze using a video behavioural analysis system provided by the Huaibei Zhenghua Biological Equipment Co., Ltd. (ZH-3000 type, Anhui, China). After completion of adaptive training, diet was restricted to 85% of ad libitum daily consumption by weight. The rats were placed in the central area of a maze with an opaque cover, and the arm doors were opened. Behaviour was recorded for 10 min. Rats entered each arm from the central area searching for food, and entering an arm was considered to have occurred when 70% of the body was inside the door to the arm. If the food had not been finished after 10 min, the test was terminated. Rats were tested once daily for 5 days. Working memory errors, reference memory errors, and total foraging time were recorded.

### Plasma Aβ42 and indicators of glucose metabolism

Before plasma sampling, rats were fasted for 12 h with water available. Blood (0.5 mL) was then collected from the retinal venous plexus and centrifuged (5,000 rpm, 10 min) to separate the plasma. Plasma glucose was determined by the glucose oxidase method using a kit (Nanjing Jiancheng Technology Co., Ltd., Nanjing, China). Plasma insulin and Aβ42 levels were measured by ELISA using a kit (Bluegene Biotech Co., Shanghai, China).

### Statistical analysis

SPSS 21.0 for Windows was applied for statistical data processing, and results were expressed as means ± standard deviations. Groups were compared using the independent-samples *t* test. The Pearson correlation test was used for evaluation of relationships between the various biomarkers (ACh, Aβ42, P-tau, IL-6, insulin, glucose, and other indicators). *P* < 0.05 was adopted as the significance level for both *t*- and Pearson tests.

## Results

### Weight and general characteristics observed in diabetic rats

**Fig. 1:**
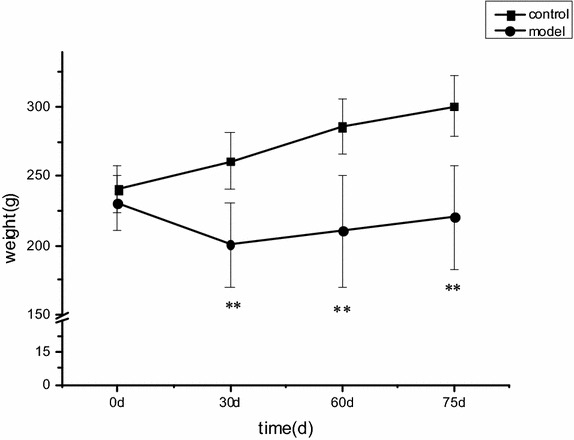
Dynamic changes in rat body weight. In this figure, model rats (streptozotocin-treated group, n = 15) are compared with control rats (control group, n = 12). Means ± standard deviations are shown. For intergroup comparisons: *P < 0.05, **P < 0.01.

Compared with the control group, rats in the experimental diabetic model group showed apathy; curled, loose stools; wet litter; a heavy smell of urine; and other phenomena. Seventy-two hours after intraperitoneal injection of STZ, the rats exhibited overeating, over drinking, and polyuria, a syndrome consistent with the clinical symptoms of diabetes. As in human diabetes, weight loss was observed (Fig. 1).

### Fasting plasma glucose and insulin levels in diabetic rats

**Fig. 2:**
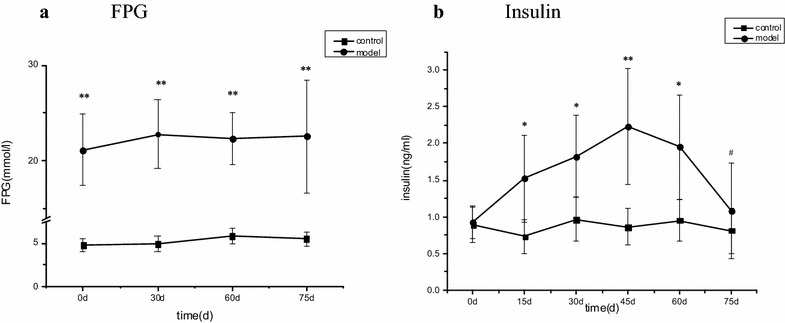
Dynamic changes in **a** fasting plasma glucose and **b** plasma insulin. In these figures, model rats (streptozotocin-treated group, n = 15) are compared with control rats (control group, n = 12). Mean ± standard deviation is shown. For intergroup comparisons: *P < 0.05, **P < 0.01; comparisons within diabetic group between time points: ^#^ *P* < 0.05.

Our study found that 43 of 50 rats in experimental diabetic model group had elevated blood glucose levels, defined as higher than 16.7 mmol/L, at 72 h (0 day); these rats were incorporated into the experiment. In the remaining animals, four died and three showed blood glucose levels not significantly changed. Compared with the control group, glucose was significantly increased in the experimental diabetic model group at day 30, day 60, and day 75 (*P* < 0.01). Insulin in plasma was elevated in diabetic rats at day 15, day 30, day 45, and day 60 (*P* < 0.05 or 0.01); However, at day 75, plasma insulin in diabetic rats was reduced compared with that in the same group at day 45 (*P* < 0.05) (Fig. 2).

### Dynamic changes in Aβ42, P-tau, IL-6, and ACh levels in CSF of diabetic rats

**Fig. 3:**
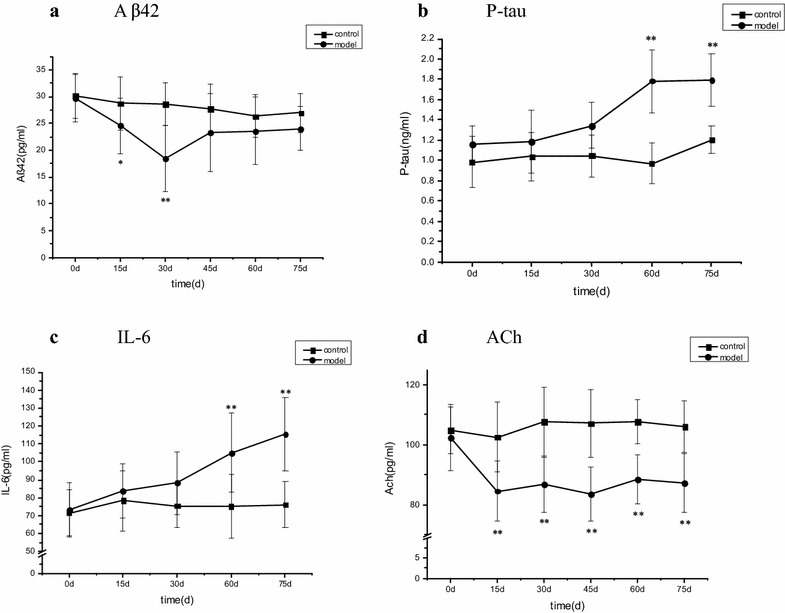
Dynamic changes in CSF levels of **a** Aβ42, **b** P-tau, **c** IL-6, and **d** ACh of cognitive impairment. In these figures, model rats (streptozotocin-treated group, n = 15) are compared with control rats (control group, n = 12). Means ± standard deviations are shown. For intergroup comparisons: *P < 0.05, **P < 0.01.

During the experiment, CSF was collected six times from diabetic rats over the period day 0 to day 75. Compared with the control group, Aβ42 was lower in the CSF of diabetic rats at day 15 and at day 30 (*P* < 0.05 or 0.01, respectively); it was also lower at day 30 compared with previous levels in the same group at day 15 (*P* < 0.01). Model levels were also higher at day 45, day 60, and day 75 than in the same group at day 30 (*P* < 0.01) (Fig. 3a). Compared with the control group, P-tau levels in the CSF of the experimental diabetic model group were significantly increased at day 60 and day 75 (*P* < 0.01) (Fig. 3b). IL-6 was also increased in the CSF of the model group at day 60 and day 75 (*P* < 0.01) (Fig. 3c). Compared with the control group, ACh in the CSF of the diabetic rat model was severely lowered at day 15 (*P* < 0.01), continuing so to day 75 (Fig. 3d).

### Determination of Aβ42 content in the blood of diabetic rats

**Fig. 4:**
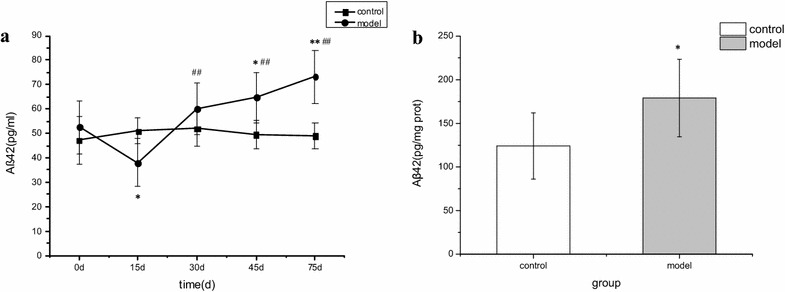
**a** Dynamic changes of Aβ42 in plasma of diabetic rats. In these figures, model rats (streptozotocin-treated group, n = 15) are compared with control rats (control group, n = 12). Mean ± standard deviation is shown. For intergroup comparisons: *P < 0.05, **P < 0.01; comparisons within diabetic group between time points: ^##^ *P* < 0.01. **b** Brain tissue Aβ42 concentrations at day 75 (n = 10–12). For intergroup comparisons: *P *<* 0.05.

Compared with the control group, Aβ42 in the blood of the diabetic rats was significantly lower at day 15 (*P* < 0.05) and significantly higher at day 45 and day 75 (*P* < 0.05 and 0.01, respectively). The Aβ42 levels in the blood of diabetic rats at day 30, day 45, and day 75 were significantly increased compared with those in the same group at day 15 (*P* < 0.01). The Aβ42 levels in the blood of diabetic rats at day 75 were also significantly increased compared with those at day 30 (*P* < 0.05) (Fig. 4a).

### Determination of Aβ42 content in the brain tissue of diabetic rats

Compared with the control group, Aβ42 content in the brain tissue of diabetic rats was found to be increased by day 75 (178.99 ± 44.47 pg/mg prot; *P* < 0.05) (Fig. 4b).

### Correlation analysis of various biomarkers in diabetic rats

Pearson correlation analysis showed that changes in ACh levels showed a high negative correlation with Aβ42, P-tau, and IL-6 in the CSF of diabetic rats, *r* = −0.916 (*P* < 0.05). In addition, at day 0 of the diabetes model, the indicators were not correlated; at day 15 of the model, Aβ42 in CSF showed a strong negative correlation with blood glucose, *r* = −0.865 (*P* < 0.05). At day 45 of the model, Aβ42 in CSF was strongly negatively correlated with insulin, *r* = −0.760 (*P* < 0.05) and insulin resistance index, *r* = −0.946 (*P* < 0.05). At day 75, Aβ42 in the blood and brain in the experimental diabetic model group showed low correlation (Tables 1, 2).

**Table 1:** Pearson correlation of diabetes biomarkers with cognitive impairment biomarkers in diabetic rats

	FPG					Insulin					IRI				
	Day 0	Day 15	Day 30	Day 45	Day 75	Day 0	Day 15	Day 30	Day 45	Day 75	Day 0	Day 15	Day 30	Day 45	Day 75
CSF Aβ42	−0.238^NS^	−0.865*	−0.548^NS^	−0.343^NS^	−0.720^NS^	−0.267^NS^	0.256^NS^	0.074^NS^	−0.760*	−0.372^NS^	−0.388^NS^	0.064^NS^	−0.443^NS^	−0.946*	−0.625^NS^
Blood Aβ42	0.257^NS^	0.744^NS^	0.497^NS^	−0.767^NS^	−0.002^NS^	−0.742^NS^	0.669^NS^	−0.790^NS^	−0.036^NS^	−0.411^NS^	−0.481^NS^	0.365^NS^	−0.151^NS^	−0.869^NS^	−0.130^NS^
ACh	0.128^NS^	0.137^NS^	0.101^NS^	−0.079^NS^	−0.347^NS^	0.028^NS^	−0.032^NS^	−0.490^NS^	−0.009^NS^	−0.608^NS^	0.338^NS^	0.256^NS^	−0.310^NS^	0.888^NS^	−0.874^NS^

The Pearson correlation test was used for evaluation of relationships between the various biomarkers (n = 25). *P* < 0.05 (*) was adopted as the significance level for all tests. *P* > 0.05 (^NS^) was not considered significant.

*CSF Aβ42* amyloid β42 in cerebrospinal fluid, *Blood Aβ42* Aβ42 in blood, *IRI* insulin resistance index.

**Table 2:** Correlation analysis of Aβ42 in blood and cerebrospinal fluid in diabetic rats

Time point	Pearson correlation	*P* value
Day 0	0.327^NS^	0.295
Day 15	−0.647^NS^	0.174
Day 30	−0.623^NS^	0.283
Day 45	0.604^NS^	0.140
Day 75	−0.693^NS^	0.256

The Pearson correlation test was used. *P* < 0.05 (*) was adopted as the significance level for all tests. *P* > 0.05 (^NS^) was not considered significant. Results are shown for day 0 to day 75 in diabetic model rats (n = 15).

### Determination of learning and memory ability by the eight-arm maze test in diabetic rats

Foraging time was significantly longer in diabetic rats compared with control (*P* < 0.05). The number of reference memory errors was also increased significantly in diabetic rats compared with control (*P* < 0.05), suggesting that learning and memory ability in the diabetic rats were damaged by day 71 (Table 3).

**Table 3:** Learning and memory capacity in diabetic rats at day 71—eight-arm maze test

Group	n	Foraging time (s)	Number of reference errors
Control	10	366.5 ± 64.8	2.78 ± 0.33
Model	15	505.5 ± 53.3*	5.40 ± 0.85**

A rat diabetes model (streptozotocin-treated group, n = 15) is compared with control (n = 10). Means ± standard deviations are shown. For intergroup comparisons: *P < 0.05, **P < 0.01.

## Discussion

With the rapid recent increase in the incidence of diabetes, cognitive impairment induced by diabetes has attracted wide attention [1]. Since 2006, when Mijnhout [12] proposed a ‘diabetes-related cognitive impairment’, several studies have shown that diabetic patients and animal models exhibit cognitive dysfunction. Takeda’s research deepened understanding of the mechanistic links between the two types of disease [13]. Recent studies have shown unimpaired cognition in the AD process at the time when Aβ deposition occurs, and only after a delay are neurological dysfunction and degeneration seen [14]. We therefore have reason to believe that the brain levels of Aβ, P-tau protein, IL-6, and ACh mark early events in memory impairment.

From previous literature, it appears that there is no standard cognitive dysfunction induced by diabetic animal models; usually a DM model was established using a drug, and with extension of modelling time, cognitive function was observed to decline. The present study uses a single intraperitoneal injection of STZ to establish the model and tests were conducted over 75 days. During the experiment, the blood glucose in the experimental diabetic model group was stable at above 16.7 mmol/L, showing good model stability.

In this study, the various biomarker changes were followed by collecting CSF from the rats, methods for which are broadly divided into two categories: the lateral ventricle puncture, involving drilling a hole in the animal’s skull and inserting a drainage tube for collecting cerebrospinal fluid [15], and the extraction of CSF via a needle puncture into the cisterna magna [11]. Our experiment used a method based on a previous publication describing a one-time technique for collection through an intravenous infusion needle. We slightly modified this to a technique for multi-time-point collection from a single animal. This enabled us to collect CSF from diabetic rats longitudinally over 0–75 days. This allowed us to avoid the need to sacrifice animals at each time point, thereby reducing the standard deviation of the data. As a result, dynamic biomarker changes were observed when DM caused major cognitive damage. Aβ42 and ACh in brain were the first to change significantly; P-tau and IL-6 protein concentrations changed later and in a more gradual, progressive manner. Currently, there are reports of longitudinal clinical studies of biomarkers of cognitive impairment; however, previous studies tended to explore more than a single pathology, rarely also discussing two or more dynamic changes in biomarkers or the long-term pathological evolution (e.g., over 2 months). In the present study, we collected cerebrospinal fluid from the cisterna magna five or six times per animal at several time points. The amount of CSF that could be collected per animal per time point was limiting in this experiment because of practical issues with diabetic rats; therefore, not all the indicators were measured at some time points. However, from related references and pilot experiments, we knew that diabetic rats have a cognitive impairment detectable from day 60 to day 70, and therefore, we knew the appropriate time to terminate the experiment and collect larger, adequate samples from all the animals. Before and after data were therefore available for all biomarkers. Because of the abovementioned limitation, our laboratory intends to perform studies using non-invasive CSF collection methods, seeking methods by which it will be practical to collect cerebrospinal fluid at shorter time intervals, allowing better data to be obtained in future experiments.

The prevailing view is that diabetes is closely related to brain aging and dementia. Dementia is thought to result from an imbalance between Aβ production and clearance, leading to loss of neurotransmitter expression and damaged synaptic function [16, 17]. This study found at day 15 a strong negative correlation of CSF levels of Aβ42 with blood glucose. Accordingly, we have reason to believe that, with the establishment of the diabetes model at day 0–day 15, elevated blood glucose can cause a severe precipitation phenomenon in Aβ42 in vivo, Aβ42 in CSF showing a decline in the early stages of diabetes. It is known that Aβ42 levels in CSF inversely reflect the brain content of the fibrous, insoluble form of Aβ, which is the core component of senile plaques and is important in their formation. Its precipitation occurs early in AD [18, 19]. In our model, the interval 15–30 days, when changes in blood glucose had stabilized, was instructive. Changes in Aβ42 levels in CSF bottomed later than did those in the blood, and we therefore speculate that at this time Aβ deposition may begin to appear in the brain. At day 30, Aβ42 in CSF hits ‘rock bottom’ due to the double impact of deposition and glycosylation.

It has been reported that hyperinsulinaemia in patients with chronic insulin resistance carries a significantly increased risk of cognitive dysfunction or dementia [20]. Watson et al. found that in healthy older people who inject insulin, both insulin level and Aβ42 level could be increased in CSF [21]. In the present study, with synchronous dynamic monitoring of plasma insulin levels in diabetic rats, we found that when the model plasma insulin levels were increased vs. controls (day 30–day 45), a peripheral insulin resistance phenomenon being dominant, the plasma Aβ42 level was also significantly increased vs. controls, presumably due to Aβ42 degradation and insulin degradation competing for the same substrate—insulin-degrading enzyme [22, 23]. In the diabetes model, day 45–day 75 is a period of falling plasma insulin levels, which may be associated with a negative feedback mechanism that reduces insulin levels by up-regulating expression of insulin-degrading enzyme, accelerating Aβ42 degradation as well. However, the enzyme was again inhibited, leading to elevated blood levels of Aβ42. At the same time, correlation analysis at diabetic model day 45 showed Aβ42 in CSF to be negatively correlated with plasma insulin and insulin resistance index. By observing changes over time, it can be seen that day 45 is the watershed of insulin level change, marking the beginning of a decrease in insulin levels. At this time point, cerebrospinal fluid Aβ42 levels began to rise, the opposite trend, showing a negative correlation.

It is known that P-tau concentration reflects the level of neurofibrillary tangles in the brain. This study found that changes in P-tau in the CSF of rats in our diabetic model group occurred later than the changes in Aβ42; a significant increase in P-tau in CSF was observed only at day 60 and day 75. Studies have shown that after a fall in peripheral insulin levels, the insulin signalling pathway is down-regulated, resulting in the downstream phosphatidylinositol 3-kinase activity being decreased while the PI-3K activity is decreased, which will cause downstream tau protein phosphorylation [24]. The main kinase, glycogen synthase kinase-3β, passes from a non-activated to an activated state. Thus, there will be hyperphosphorylation of tau protein leading to AD-like changes [25]. Therefore, we speculate that abnormal PI-3K pathway activation of glycogen synthase kinase-3β in an era of declining insulin caused tau protein phosphorylation to be increased in our model.

Analysis shows that the trajectory changes were different over time. In diabetic rats at each time point, fluctuations in Aβ42 and P-tau protein concentration in CSF appeared complementary, namely ‘shift’ changes over time. P-tau protein was on a gradually increasing trend when Aβ42 was decreased relative to controls, which is consistent with clinical reports [26]. The experiment found that in individual diabetic animals, lower Aβ42 in CSF at early times (day 15–day 30 time-points) appears to reduce the phenomenon of phosphorylated tau protein. The experiment also found that IL-6 expression at model day 60–day 75 was increased (*P* < 0.01), plasma Aβ42 at model day 15 was decreased (*P* < 0.01), and Aβ expression in rat brain tissue at model day 75 was increased (*P* < 0.05). These results are consistent with previous reports [27].

Currently, intraperitoneal injection of STZ to induce learning and memory dysfunction in rodent models has academic recognition [28]. In agreement with this, the present study found that in the diabetes model at day 71, foraging time in the eight-arm maze was significantly longer than in control rats. Our results suggest that changes in the measured biomarkers seem to appear earlier than learning and memory impairments since according to the work of others cognitive deficits do not appear earlier than 70 days after diabetes induction [29].

The above the trajectory analysis of biomarker change shows that reduced levels of Aβ42 in CSF is an early event, and that P-tau protein and IL-6 changes are later events. The above metabolic changes together determine the cognitive dysfunction in diabetic rats, and our study supports the hypothesis previously proposed, namely that changes in brain Aβ42, P-tau protein, IL-6, and ACh expression are the early events in diabetic memory impairment, and the latency period of the latter may provide an intervention time window for preventing cognitive impairment in DM. Moreover, deposition of brain Aβ42 predicts a cognitive-impairment pathological process, and leads to synaptic dysfunction, culminating in cell loss and brain atrophy. Cognitive impairment induced by diabetes is characterized by a diversity of mechanisms, which dictates multi-target intervention strategies. At the same time, changes in the trajectory of different biomarkers at different stages of cognitive impairment due to the different mechanisms of injury determine drug selection. Another purpose of this experiment was to study drug intervention strategies. Correlation analysis showed that ACh, Aβ42, P-tau, and IL-6 levels in the CSF of diabetic rats were not linearly related (*P* > 0.05), while further data analysis showed that ACh change and Aβ42, P-tau, and IL-6 were highly negatively correlated, suggesting that changes in ACh in diabetic rats were determined by the common factors in Aβ42, P-tau, and IL-6 variation. In the diabetes model at day 0–day 30, P-tau and IL-6 are not a significant source of variation, Aβ42 and ACh being the main factors changing. Aβ42 content in CSF was reduced, reaching a minimum at day 30. In light of the above analysis, the results suggest that in terms of choice of drug intervention strategies, blood glucose should be controlled to rectify the early Aβ42 protein deposition, etc., due to the double impact of glycosylation and insulin elevation, thus preventing possible cognitive damage. This study also found increases in P-tau and IL-6 protein in CSF in the diabetic model at day 60–day 75, but by this time Aβ42 changes had stabilized. At this stage, it is believed that the main factors causing cognitive impairment effects are decreased ACh content and increased P-tau protein and IL-6 in CSF. Based on the above considerations, targeted intervention therapy with drugs to ameliorate insulin resistance and combat inflammation should be considered the best choice for this latter stage.

This study has some limitations, such as the lack of effective mathematical methods for studying the quantitative characteristics of the biomarkers. In future, we intend to use mathematical methods to reveal the quantitative relationships among the various markers and the dependence of each marker on time. Moreover, the study did not use a model appropriate to proving the effectiveness of specific drug intervention strategies. In subsequent experiments, these problems will be further addressed.

## Conclusions

We found that the trajectories of Aβ42, P-tau, IL-6, and ACh levels in CSF were different in dissimilar stages of diabetes-accelerated memory dysfunction in rats. Results from our longitudinal research model suggest that changes in the measured biomarkers appear before learning and memory impairments do. In the diabetes model group, Aβ42 and ACh changed obviously from day 0 to day 45, after which P-tau and IL-6 varied significantly from day 45 to day 75. In our diabetes model, Aβ42 in CSF may be correlated with blood glucose in rats from day 0 to day 45, after which it correlates strongly with insulin. The reduced ACh levels observed possibly correlated with the factors common to changes in Aβ42, P-tau, and IL-6.

## Abbreviations

Aβ42: amyloid β42
P-tau: phosphorylated tau protein
IL-6: interleukin 6
ACh: acetylcholine
AD: Alzheimer disease
DM: diabetes mellitus
STZ: streptozotocin
CSF: cerebrospinal fluid
